# 20-year neurocognitive development following a schizophrenia spectrum disorder and associations with symptom severity and functional outcomes

**DOI:** 10.1017/S0033291724000096

**Published:** 2024-07

**Authors:** Marie Starzer, Helene Gjervig Hansen, Carsten Hjorthøj, Nikolai Albert, Kathryn E. Lewandowski, Louise Birkedal Glenthøj, Merete Nordentoft

**Affiliations:** 1 Copenhagen Research Center for Mental Health – CORE, Mental Health Center Copenhagen, Mental Health Services in the Capital Region, Copenhagen, Denmark; 2 Department of Clinical Medicine, University of Copenhagen, Copenhagen, Denmark; 3 Department of Public Health, University of Copenhagen, Section of Epidemiology, Copenhagen, Denmark; 4 Mental Health Centre Amager, Mental Health Services in the Capital Region, Copenhagen, Denmark; 5 Schizophrenia and Bipolar Disorder Program, McLean Hospital, Belmont, MA, USA; 6 Department of Psychiatry, Harvard Medical School, Boston, MA, USA; 7 Department of Psychology, University of Copenhagen, Copenhagen, Denmark

**Keywords:** cognitive function, early specialized intervention services, first-episode psychosis, longitudinal study, long-term follow-up studies, OPUS, schizophrenia

## Abstract

**Background:**

Cognitive deficits are a core feature of schizophrenia and are closely associated with poor functional outcomes. It remains unclear if cognitive deficits progress over time or remain stable. Determining patients at increased risk of progressive worsening might help targeted neurocognitive remediation approaches.

**Methods:**

This 20-year follow-up study examined neurocognitive outcomes of 156 participants from the OPUS I trial. Neurocognition was assessed using the brief assessment of cognition in schizophrenia at the 10- and 20-year follow-up, allowing us to examine changes in neurocognition over ten years.

**Results:**

We found that 30.5% of patients had a declining course of neurocognition, 49.2% had a stable course of neurocognition and 20.3% experienced improvements in neurocognition. Good cognitive functioning at the 20-year follow-up was significantly associated with higher levels of social functioning (B 6.86, CI 4.71–9.02, *p* < 0.001) while increasing experiential negative symptoms were significantly correlated to cognitive worsening (PC-0.231, *p* = 0.029). Younger age at inclusion (B: 0.23 per 10-years, CI 0.00–0.045, *p* = 0.047) and low level of education (below ten years) (mean difference: −0.346, CI −0.616 to −0.076, *p* = 0.012) predicted declining neurocognition.

**Conclusion:**

Our findings support the notion of different schizophrenia subtypes with varying trajectories. Neurocognitive impairment at the 20-year follow-up was associated with other poor outcomes, highlighting the importance of treatments aimed at improving neurocognition in patients with schizophrenia spectrum disorders.

## Introduction

Schizophrenia is an often long-lasting and debilitating illness with recovery rates ranging from 14 to 32 percent (Hansen et al., 2023a; Jääskeläinen et al., 2013; Lally et al., 2017). Apart from psychotic and negative symptoms, patients often also display significant cognitive impairments, which are associated with poor social and vocational function (Cowman et al., 2021). Back in the 1960ties, the introduction of antipsychotic medication revolutionized the treatment of psychotic symptoms. Still, antipsychotics show minimal effects on improving cognitive deficits (Guilera, Pino, Gómez-Benito, & Rojo, 2009). Different psychosocial interventions have been developed to improve symptom levels and functioning (Bighelli et al., 2021). These interventions show great effects whilst ongoing (Bertelsen et al., 2008). Still, long-term cognitive deficits, negative symptoms and low functioning levels remain a debilitating reality for many patients with schizophrenia (Halverson et al., 2019; Silberstein & Harvey, 2019). This is well illustrated by the fact that recovery rates today do not differ greatly from those predating the neuroleptic era (Hansen et al., 2023a). Cognitive remediation has emerged as a targeted intervention for cognitive deficits in schizophrenia. Still, despite the established efficacy of cognitive remediation in improving cognitive and functional outcomes (Wykes, Huddy, Cellard, McGurk, & Czobor, 2011; Lejeune, Northrop, & Kurtz, 2021), its inclusion within established intervention programs for schizophrenia remains limited.

Two models pertaining to the basic nature of cognitive deficits in schizophrenia have been proposed (Bora & Murray, 2014). In the neurodevelopmental model, cognitive impairments arise in the premorbid phase of the illness and around illness onset, after which cognition remains stable through the remaining course of the illness. In the neurodegenerative model, cognition continues to decline after illness onset more rapidly than in healthy individuals. In addition to these models, it has also been suggested that some patients may recover and return to premorbid cognitive functioning after some time (Hedman, van Haren, van Baal, Kahn, & Hulshoff Pol, 2013).

Previous research has illustrated that cognitive decline occurs in the prodromal phase of illness, before actual psychosis onset (Mollon & Reichenberg, 2018; Fusar-Poli et al., 2012). There is, however, conflicting evidence as to how further changes in cognition occur over time, and it is uncertain if cognitive function remains stable in the later phase of illness. Most studies conducted on patients with first-episode psychosis have short-term follow-ups ranging from 1–6 years and generally report a stable course of neurocognition (Bozikas & Andreou, 2011; Rajji, Miranda, & Mulsant, 2014; Szöke et al., 2008). However, findings have been mixed with the continued decline (Barder et al., 2013; Habtewold et al., 2020; Pukrop et al., 2006), and some showing improvement in some areas (Murillo-García et al., 2023). Long-term studies with more than ten years of follow-up are limited but do generally report a declining course of neurocognition in schizophrenia (Fett et al., 2020; Jonas et al., 2022). Often, these studies suffer from small sample sizes (Fett, Reichenberg, & Velthorst, 2022; Tschentscher et al., 2023). While indirect, findings from cross-sectional studies in schizophrenia patients at different phases of illness tend to report significantly greater cognitive impairment in patients who have been ill for many years, (Bilder et al., 1992; Dickinson, Ragland, Gold, & Gur, 2008; Seidman, Buka, Goldstein, & Tsuang, 2006), compared to those experiencing an initial episode (Bora & Pantelis, 2015; Pantelis et al., 2009; Seidman et al., 2010), these cross-sectional studies might be flawed by selection bias. Some studies have tried to determine predictors of deteriorating or changing neurocognition, but results have been inconsistent (Ayesa-Arriola et al., 2023; Fett et al., 2020; Hoff, Svetina, Shields, Stewart, & DeLisi, 2005; Luther et al., 2020; Rund et al., 2016; Zanelli et al., 2019).

The current literature on long-term changes in neurocognition and its association with clinical and functional outcomes is scarce and predominantly comprises small study populations. This study aimed to extend the evidence on the long-term development of cognition in schizophrenia.

The aim of our study was to (1) determine changes in neurocognitive function over a ten-year period after initial diagnosis and to examine possible correlations with changes in symptom severity and social functioning, (2) to investigate 20-year cognitive outcomes and to determine associations with other long-term outcomes, and (3) to examine if specific baseline characteristics would predict changes in neurocognition over ten years.

## Method

### Study design and participants

This study reassessed participants from the OPUS I randomized clinical trial 20 years after the first inclusion. The original trial was conducted from 1998 until 2000 and included 578 participants with an incident schizophrenia spectrum diagnosis (ICD-10 classification: F20-F25, F28-F29). All participants had to be between the ages of 18 and 45 years and could not have received more than 12 weeks of consecutive anti-psychotic treatment before inclusion. Participants were randomized to specialized early intervention treatment (OPUS) comprised of assertive community treatment, family involvement and psychoeducation (n 275), prolonged rehabilitation (n 31), or treatment as usual (n 272) (Bertelsen et al., 2008; Petersen et al., 2005a). After two years, all patients were reassessed, and participants receiving OPUS treatment were transferred to treatment as usual. All participants were subsequently invited to participate in 5-, 10- and 20-year follow-up reassessments and provided written informed consent before each follow-up. Participants were assessed using semi-structured interviews conducted by independent clinical staff blinded to the original treatment allocation. Regular interrater sessions were conducted to secure high interrater reliability. We included 174 in the 20-year reassessment; 113 were diagnosed with schizophrenia at baseline, and 67% of these patients also qualified for schizophrenia diagnosis assessed using SCAN at the 20-year follow-up. Thirty-one were diagnosed with schizotypal disorder at baseline, and 30 were diagnosed with other psychosis at baseline (F22 – F29); 56% of these patients qualified for a schizophrenia diagnosis at the 20-year follow-up. The main sample included in this paper comprised 156 participants who had completed the full cognitive assessment battery at the 20-year follow-up, while in total, 160 had completed parts of the cognitive assessment. For the analysis of change in cognition over time, we were able to include data on 129 participants who had completed the full cognitive test battery at both the 10- and 20-year follow-up, while a total of 140 participants had repeated measurements for any of the individual cognitive domains at both follow-up points.

### Measures

#### Main outcome

**Figure 1.**
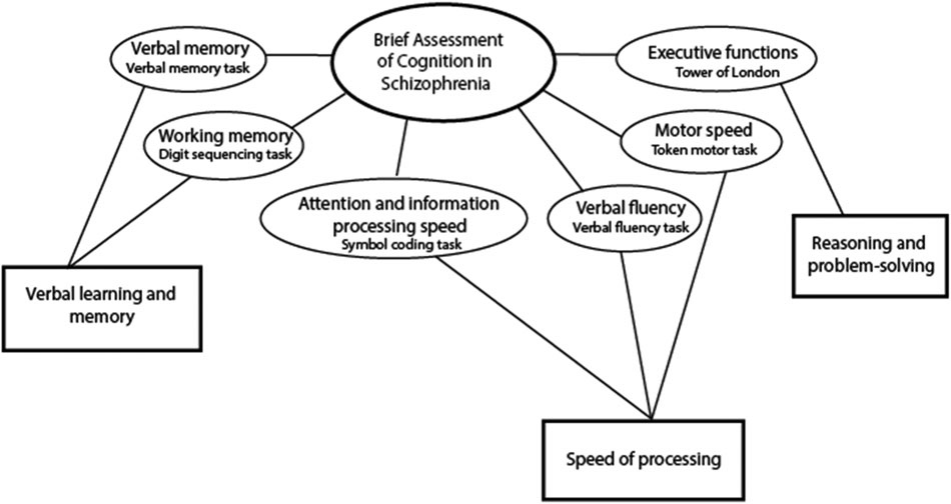
Neurocognitive subdomains - BACS three factor model (Keefe et al., 2004).

The main outcome of this study was cognitive function. The brief assessment of cognition in schizophrenia (BACS) was used to index neurocognitive functioning (Bora & Murray, 2014; Keefe et al., 2004) at the 10 and 20-year follow-up. BACS-certified clinicians administered the test. The BACS comprises six subtests and generates one global measure of cognitive functioning. Factor analyses have shown that BACS fits a three-factor model (Keefe et al., 2004) indexing neurocognition in three subdomains: (1) **Verbal learning and memory** comprised of verbal memory (verbal memory task) and working memory (digit sequencing task). (2) S**peed of processing (SP)** comprised of verbal fluency (verbal fluency task), attention and information processing speed (symbol coding task), and motor speed (token motor task). (3) **Reasoning and problem-solving** comprised the executive functions assessment (Tower of London task). The three-factor model is illustrated in Fig. 1.

#### Secondary outcome measures

We used the Scale for Assessment of Positive Symptoms (SAPS) and the Scale for Assessment of Negative Symptoms (SANS) (Andreasen, Flaum, Swayze, Tyrrell, & Arndt, 1990) to assess positive and negative symptoms. For both dimensions, we calculated composite scores ranging from 0–5. The ‘psychotic dimension’ was the mean score of the global ratings for hallucinations and delusions. The ‘negative dimension’ was the mean score of four global ratings of negative domains in SANS (Arndt, Andreasen, Flaum, Miller, & Nopoulos, 1995), excluding the attention domain. A symptom score above two on all global ratings of symptoms was to be clinically significant symptoms (Andreasen et al., 2005). We also used a two-factor model for negative symptoms, looking at experiential symptoms (comprised of anhedonia and avolition SANS scores) and expressive symptoms (comprised of affective flattening and alogia SANS scores) (Foussias & Remington, 2010; Strauss et al., 2013).

At the 20-year follow-up global level of functioning was assessed using the Global Assessment of Functioning scale (GAF-F) ranging from 0–100. Current psychiatric diagnoses and substance use disorders were assessed using the schedule for clinical assessment in neuropsychiatry (SCAN 2.1) (Schützwohl, Kallert, & Jurjanz, 2007; Wing, Sartorius, & Üstun, 1998). Information on a variety of clinical characteristics such as living conditions and housing situation, partnership situation and children, educational level, employment and workability, current psychiatric treatment, treatment with antipsychotics, suicidal ideations, quality of life, self-perceived symptoms and self-rated health were assessed in semi-structured interviews and using questionnaires (the WHO Quality of Life-BREF (WHOQoL-Bref) (The WHOQOL Group, 1996; von Knorring, 2001), the EQ-5D (Brooks, 1996), the Danish Sundhedsprofilen).

At baseline, pre-morbid social and academic functioning was assessed using the Pre-morbid Adjustment Scale (Brill, Reichenberg, Weiser, & Rabinowitz, 2008) and duration of untreated psychosis using the Interview for Retrospective Assessment of Onset of Schizophrenia (IRAOS) (Häfner et al., 1992).

At the 10- and 20-year follow-up, social functioning was measured using the Personal and Social Performance Scale (PSP) (Tegeler & Juckel, 2007). This scale measures four domains of social functioning: useful activities, personal relationships, self-care, aggressive and disturbing behavior – combined and assessed on a scale ranging from 0–100.

### Predictors of neurocognitive change

To establish stronger evidence for an association between baseline clinical characteristics and changes in cognitive function over time, we wanted to cross-validate predictors of cognitive outcomes already identified in other studies. The choice of baseline predictors of cognitive development was based on a systematic literature search of cross-sectional and longitudinal predictors of cognition in FEP identified in previous studies. The search strategy and selection process are displayed in Appendix A. Variables associated with long-term neurocognition were: (1) sex, (2) age, (3) age at illness onset, (4) employment status, (4) level of education (above/below ten years) (5) premorbid academic functioning and (6) schizophrenia diagnosis, (7) early specialized intervention treatment (8) cannabis cessation after the 10-year assessment, and (9) stable symptom remission within the first year after diagnosis.

### Statistical analysis

All analyses were conducted using SPSS version 25.

The raw scores of all neurocognitive subdomains were normally distributed, except for the Tower of London score assessing reasoning and problem-solving. Because of the skewness in this domain, z-scores lower than three standard deviations below the mean were recoded into −3. For cross-sectional analysis at the 20-year follow-up, we calculated Z-scores for all neurocognitive domains using the mean and s.d. of the sample (*N* = 156) at 20 years. We calculated 10- and 20-year Z-scores for longitudinal analysis using the mean and s.d. from the 10-year follow-up. Z-scores for all functions were combined into one composite z-score for BACS, termed the global neurocognitive score. Z-scores from the verbal fluency task, the symbol coding task and the token motor task were combined into a subdomain Z-score for speed of processing and Z-scores from the verbal memory task, and the digit sequencing task was combined into a Z-score for verbal learning and memory. To determine if 20-year-neurocognitive scores were associated with other 20-year outcomes, we used linear regression analysis for numerical outcomes and binary logistic regression for categorical variables.

#### Longitudinal analyses

We used paired *t* tests to assess significant differences in participants levels of neurocognition and symptoms from 10 to 20 years. Change scores were calculated for all neurocognitive domains by subtracting the 10-year from the 20-year Z-scores. Pearson correlation analyses were used to examine correlations between neurocognitive change-scores and change-scores of symptom dimensions and social functioning from the 10- to 20-year follow-up.

To determine change-scores we subtracted the 10-year Z-scores from the 20-year Z-scores. We used univariable and multivariable ANCOVA analysis to assess statistically significant predictors of neurocognitive change scores. All tests were two-tailed. Significant variables from the univariable analysis (significance set at *p* = 0.1) were included in a multivariable model using a backward elimination approach and set significance level at *p* = 0.05. We also divided participants into three groups based on these change scores, one group representing patients with improving neurocognition with change scores >0.5 s.d., one group representing participants with stable neurocognition with changes scores between 0.5; and −0.5 s.d., and one group representing patients with declining neurocognition with change scores <-0.5 s.d.

## Results

### Drop-out analysis

At the 20-year follow-up, we reassessed 174 participants, and 156 completed the full neurocognitive test battery. Due to the notable attrition rate in our sample, a drop-out analysis was performed to investigate significant differences between participants and non-participants at baseline. The results, presented in online Supplementary eTable 2 in Appendix B, revealed that participants in this study were younger (25.7 (s.d. 5.7) *v.* 26.9 (s.d. 6.5) *p* = 0.021), and there was a lower proportion of men (48.7% *v.* 63,3% *p* = 0.002) among the participants, compared to non-participants. Additionally, participants had higher levels of education (43.6% *v.* 29.7% *p* = 0.002), a higher prevalence of schizotypal disorder (19.2% *v.* 12.3% *p* = 0.043), a significantly higher level of functioning (GAF 44.2 [s.d. 13.2] *v.* 39.7 [s.d. 13.1] *p* < 0.001) and lower levels of negative symptoms (1.9 [s.d. 1.1] *v.* 2.3 [s.d. 1.2], *p* = 0.001) compared to non-participants at baseline. An additional analysis of significant differences in 10-year cognitive function between participants and non-participants in the 20-year follow-up also revealed that participants in the 20-year reassessment had better 10-year cognition than non-participants (mean 0.24 s.d. 1.33 *v.* mean −0.17 s.d. 0.63, *p* < 0.001), results are presented in online Supplementary eTable 3.

### Long-term neurocognitive and sociodemographic outcomes

**Figure 2.**
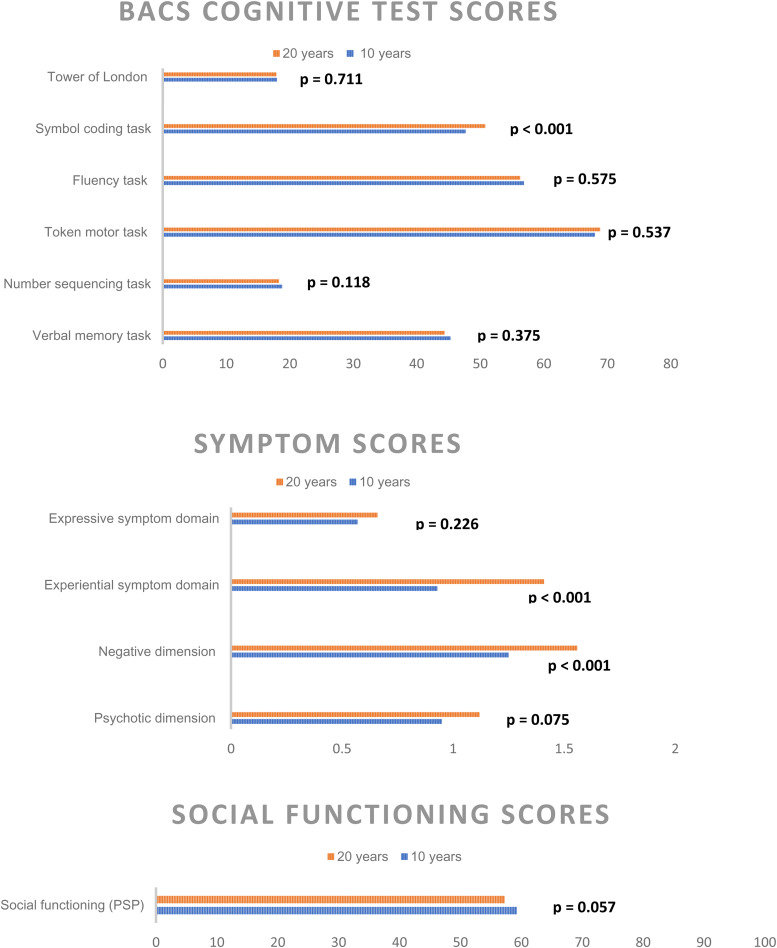
Significant difference in Cognitive BACS test raw-scores, symptom levels, and social functioning from the 10- and 20-year follow-up reassessments following a first incident schizophrenia spectrum diagnosis.

Cognitive test outcomes, symptom levels, and social functioning between the 10- and 20-year follow-up are illustrated in Fig. 2, sociodemographic data and psychopathological raw-scores are also presented in online Supplementary eTable 2 in Appendix B. With exception of the symbol coding task (mean 47.7 [s.d. 13.8] at 10-year *v.* 50.8 [s.d. 14.7] at 20-year *p* < 0.001), we found no significant differences between the two time points. Psychotic symptom scores and level of social functioning also did not differ significantly, but overall negative symptoms (1.6 [s.d. 1.1] *v.* 1.2 [s.d. 1] *p* < 0.001) and the negative symptom subdomain experiential symptoms (1.4 [s.d. 1] *v.* 1 [s.d. 0.9] *p* < 0.001) were significantly higher at the 20-year follow-up.

Sociodemographic 20-year outcomes associated with the global level of neurocognition at the 20-year follow-up, are shown in Table 1, subdomains are presented in online Supplementary eTable 4. Participants with higher neurocognitive levels were significantly more likely to be in a relationship (OR: 1.56, CI 1.1–2.2, *p* = 0.013) and be employed (OR:2.07, CI 1.35–3.17, *p* < 0.001). They were less likely to have a schizophrenia diagnosis (OR:0.59, CI 0.42–0.84, *p* = 0.003) or receive treatment with antipsychotic medication (OR:0.39, CI 0.26–0.58, *p* < 0.001). Higher global neurocognitive scores at the 20-year follow-up were also significantly associated with higher levels of social functioning (B 6.86, CI 4.71–9.02, *p* < 0.001), explaining 21% of the variance (**R^2^ 0.21)**, lower levels of psychotic symptoms (B:-0.24, CI −0.46 to −0.03, *p* = 0.027) explaining 3% of the variance (**R^2^ 0.03)** and lower levels of negative symptoms (B:-0.476, CI −0.64 to −0.32, *p* < 0.001) explaining 18% of the variance (**R^2^ 0.18)**.

**Table 1.** Analysis of 20-year clinical outcomes associated with 20-year neurocognition z-scores

Global cognition, composite BACS increasing Z-scores (n 156)			
Bivariate logistic regression of categorical outcomes			
	Odds ratio and (CI)		P-value
In a relationship	1.56 (1.1:2.2)		0.013
Employment	2.07 (1.35:3.17)		<0.001
Independent living	2.06 (0.72:5.91)		0.178
Schizophrenia diagnosis	0.59 (0.42:0.84)		0.003
Use of antipsychotic medication	0.39 (0.26:0.58)		<0.001
Linear regression analysis of numerical outcomes			
	B (95% CI)	√R	p-value
Social functioning (PSP)	6.86 (4.71:9.02)	0.21	<0.001
Psychotic symptoms	−0.24 (−0.46:-0.03)	0.03	0.027
Negative symptoms	−0.476 (−0.64:-0.32)	0.18	<0.001

### Changes in neurocognition

**Figure 3.**
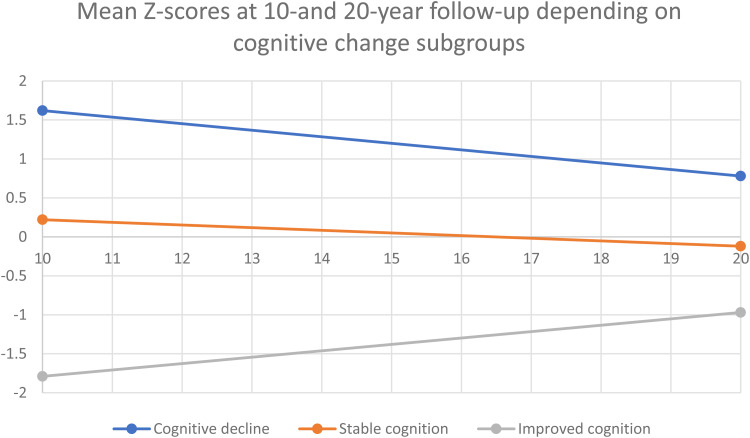
Couse of cognitive change based on subdivision of participants depending on cognitive change-scores.

Participants were divided into three groups based on their change scores. Half of the participants (49.2%) had stable global neurocognitive scores (change scores between 0.5 s.d. and −0.5 s.d. of the mean), 20.3% had improving neurocognitive scores (change scores above 0.5 s.d. of the mean), and 30.5% had deteriorating neurocognitive scores (change scores below 0.5 s.d. of the mean). Similar participant distributions were seen in all cognitive subdomains, with 25.5% showing deterioration of executive functioning, 34.6% showing deterioration of speed of processing and 34.1% showing deterioration of verbal learning and memory. Results are shown in Table 2, and Fig. 3 illustrates that participants in the cognitive decline group had higher cognitive scores at both assessments and participants in the improved cognition group had the lowest cognitive function at both time points. Age and sex distribution within the groups is shown in Table 3; the mean age in the decline group was 44.4 (s.d. 4.3); in the stable group, it was 46.5 (s.d. 6); and in the improving group, it was 47.3 (s.d. 6.1) but the difference was not significant (*p* = 0.086).

**Table 2.** Subdivision of participants depending on cognitive change-scores

	Global (n 129)		Verbal learning and memory (139)		Speed of processing (135)		Executive functioning (137)	
Grouping*	Number and (%)	Change scores Mean and s.d.	Number and (%)	Change scores Mean and s.d.	Number and (%)	Change scores Mean and s.d.	Number and (%)	Change scores Mean and s.d.
Decreasing below −0.5 s.d.	39 (30.5%)	−0.87 (s.d. 0.24)	46 (34.1%)	−0.90 (s.d. 0.27)	46 (34.6%)	1.07 (s.d. 0.59)	35 (25.5%)	0.90 (s.d. 0.69)
Stable between −0.5 and 0.5 s.d.	63 (49.2%)	−0.05 (s.d. 0.28)	74 (54.8%)	0.07 (s.d. 0.27)	62 (46.6%)	0.08 (s.d. 0.27)	62 (45.3%)	0 (s.d. 0.17)
Increasing above 0.5 s.d.	26 (20.3%)	1.07 (s.d. 0.45)	15 (11.1%)	0.94 (s.d. 0.56)	25 (18.8%)	0.90 (s.d. 0.32)	40 (29.2%)	−0.87 (s.d. 0.63)

**Table 3.** Age and sex differences depending on cognitive change subgroups

	Declining cognition (39)	Stable cognition (63)	Improved cognition (26)	*p*-Value
Age (mean and s.d.)	44.4 (4.3)	46.5 (6)	47.3 (6.1)	ANCOVA between groups *p* = 0.086
Age groups: (n and %)				
Under 40	2	3	1	Chi-square between groups *p* = 0.087
40–49	31	44	17	
50–59	6	13	7	
60 and above	0	3	1	
Female sex	22 (56.4%)	30 (52.4%)	14 (53.8%)	Chi-square *p* = 0.666

### Correlations between changes in cognitive functioning, symptom severity, and social functioning

Using Pearson Correlation analyses, we identified that increasing experiential negative symptom scores were significantly correlated to decreasing global neurocognitive scores (PC-0.231, *p* = 0.029). Results are shown in Table 4. In the subgroup analysis shown in online Supplementary eTable 5 increasing negative symptom scores were significantly associated with decreasing verbal learning and memory and speed of processing scores (R0.177, *p* = 0.038 and PC −0.192, *p* = 0.026).

**Table 4.** Correlations between change in global cognition and change in symptom severity and social functioning, Pearson correlation analysis

	Composite neurocognitive change scores (n 129)	
	Pearsons correlation and (CI)	p
Positive symptom change scores	−0.02 (−0.19:0.16)	0.858
Negative symptom change scores	0.16 (−0.02:0.32)	0.077
*- Experiential symptom change scores*	***0.23* (*0.03:0.42*)**	***0.029***
*- Expressive symptom change scores*	*−0.09* (*−0.09:0.26*)	*0*.*331*
Social functioning change scores	−0.04 (−0.22:0.13)	0.618

### Predictors of change in neurocognition

One-way ANCOVA analysis was performed to identify baseline clinical and demographic characteristics associated with long-term changes in neurocognition. In the univariate analyses, we found younger age, unemployment, lower level of education and lower premorbid academic functioning at baseline were all associated with a decline in global neurocognition. The neurocognitive subgroup analysis results are shown in eTable 4 in supplementary material Appendix B. We then conducted a multivariate ANCOVA with the significant variables identified in the univariable analyses. Only younger age at baseline (B: 0.027 per 10 years, CI 0.05–0.049, *p* = 0.016) and low level of education (below ten years) (mean difference-0.438, CI −0.694 to −0.182, *p* < 0.001) remained significantly associated with a decline in global neurocognition, results are shown in Table 5. Results of the neurocognitive subgroup analysis are shown in eTable 4 in supplementary materials.

**Table 5.** Baseline predictors of changes in global neurocognition from the 10-to 20-year follow-up

Univariate ANCOVA analysis (significance set at *p* = 0.01)		
	Change in global function (*n* 129)	
Baseline characteristics	B (95% CI)	*p*-value
Male sex	−0.127 (−0.387:-0.133)	0.336
Age (increasing)	0.019 (0.003: 0.042)	0.091
Unemployment	−0.417 (−0.679:-0.154)	0.002
Lower level of education	−0.373 (−0.628:-0.117)	0.005
Lower premorbid academic functioning	0.212 (0.429:-0.005)	0.056
OPUS	−0.005 (−0.160:0.151)	0.954
Stable remission first year	0.095 (−0.198:0.388)	0.524
Higher age at illness onset	0.014 (−0.008:0.036)	0.208
Longer duration of untreated psychosis (weeks)	0.000 (−0.001:0.001)	0.362
Schizophrenia diagnosis	−0.014 (−0.089:0.060)	0.702
Cessation of alcohol or substance use, including cannabis	0.089 (−0.531:0.708)	0.778
Multivariate ANCOVA analysis		
Age (increasing)	0.027 (0.05:0.049)	0.016
Unemployment	−0.272 (−0.547:0.003)	0.052
Lower level of education	−0.438 (−0.694:-0.182)	<0.001
Lower premorbid academic functioning	0.106 (−0.123:0.334)	0.362

In univariate analysis we found no variables associated with verbal learning and memory, speed of processing and executive functions, so we did not conduct multivariable analysis for these domains.

### Discussion

We found no significant differences between the six BACS domain scores at the 10- and the 20-year follow-up except for the symbol coding task that showed higher test scores at the 20-year follow-up. Social functioning and psychotic symptoms also remained stable, but the global level of negative symptoms, and in particular experiential negative symptoms, was higher at the 20-year follow-up. This contrasts with previous long-term follow-up studies (Fett et al., 2020; Jonas et al., 2022), generally reporting a cognitive decline. Additionally, the findings highlight expressive negative symptoms as a specific symptom aspect that may be further impaired in the long-term course of schizophrenia and may be an essential focus for targeted interventions in FEP.

#### Changes in neurocognition

Although we could not identify significant differences in neurocognitive test-scores from the 10-to the 20-year follow-up across the total sample, we determined subgroups according to BACS change-scores. We found that 49.2% of participants had a stable course of global neurocognition, while 30.5% had declining neurocognitive scores, and 20.3% showed improvement. These findings disprove neither the neurodevelopmental theory, in which cognition remains stable after the initial onset of psychosis, nor the neurodegenerative model, in which cognition declines further following the onset of psychosis, as both scenarios occur in different patients. Some patients even seem to recover cognitive functions. This emphasizes the possibility of very different outcomes in schizophrenia. The distribution of patients in these groups was similar to that reported by other long-term follow-up studies (Rodríguez-Sánchez et al., 2022; Zanelli et al., 2019), albeit some studies have reported no patients with an ongoing decline of neurocognition (Murillo-García et al., 2023; van Winkel et al., 2006). In general, the research field is dominated by conflicting evidence on the stability of cognitive function after illness onset. This may be because follow-up studies examining the period after a first-episode psychosis include mainly young patients with subtle cognitive changes hard to detect. As patients age, cognition may start to decline more rapidly and, therefore, is more easily detected. Perhaps more broad-based cognitive testing at illness onset could increase the possibility of detecting subtle deficit areas.

We found no significant differences between the groups with declining, stable or improving cognition regarding age and sex, but there was a tendency towards participants with decline to be younger. This is in contrast to a study of older chronically ill patients with schizophrenia reporting that a larger portion of patients started to display a declining course of neurocognition as age increased (Kida et al., 2020). Our study detected higher attrition at the 20-year follow-up of patients with poor cognitive function at the 10-year follow-up. Patients seemed likelier to participate in the 20-year reassessment if they had good cognitive function, especially in older patients, as they mainly belong to the continued stable or improving cognition groups. This indicates that cognitive decline might prevent participation in our follow-up because only those with a decline from high cognitive function are represented in the sample. It is possible and very worrisome that cognitive decline later during illness has been overlooked because of a systematic failure to include the most affected patients in follow-up assessments. The Suffolk County 20-year follow-up had a remarkably low drop-out rate, and they are the only large longitudinal study to show consistent and significant cognitive decline in their patient population. (Jonas et al., 2022). We found that 30% of patients experienced cognitive decline, and our findings may underestimate the rates of patients with cognitive decline in a real-world schizophrenia population. Pro-cognitive interventions in schizophrenia should not only be offered as part of early intervention services; they might also really benefit some patients in more chronic illness stages. Further research is needed to clarify this issue.

### Predictors of change in neurocognition

Our study only identified younger age at baseline and lower level of education as predictors of declining global neurocognition. That younger age at baseline predicted declining cognition in our study contrasts findings from other long-term follow-up studies (Fett et al., 2020; Meier et al., 2014). These studies indicated that the influence of increasing age on cognition became significant after a certain age had been reached, and this is further supported by the Suffolk County study reporting declining neurocognitive function in both patients and controls but a more rapid decline in patients with schizophrenia compared to individuals without schizophrenia (Jonas et al., 2022).

The finding that a lower level of education was associated with cognitive decline corroborates findings from the Spanish PAFIP 10-year follow-up (Ayesa-Arriola et al., 2023; Murillo-García et al., 2023). We could not cross-validate other predictors from previous long-term follow-up studies, perhaps because our longitudinal data lacked cognitive assessments at baseline, which limited our ability to conclude predictors of the course of cognition from illness onset. All predictors of poor outcomes in the 10-year OPUS follow-up study were included in the prediction analysis of this paper, see Appendix A. Only baseline lower level of education predicted poor cognitive function in the OPUS 10-year follow-up and cognitive decline between the 10-and 20-year follow-up. Because the sample in the 10-year follow-up was twice the size of the 20-year follow-up, we may have lacked the power to determine the same predictors. However, drop-out analysis revealed a tendency for participants with poor cognition to drop out, and we found that those with the lowest cognitive function did not decline. Rather, those with the highest cognitive function seemed to decline between the 10- and 20-year follow-up; this could also explain why predictors of poor cognitive function at the 10-year follow-up would not predict further decline, as those with poor cognition did not experience the worst decline.

It may seem especially surprising that we did not find any associations between cessation of substance use and changes in cognition. Because we used SCAN-verified diagnosis of substance use as the only measure of substance use, this very likely led to the exclusion of participants with recreational use from the analysis, and perhaps we did not find an association because our definition was too narrow and the sample too small. Alcohol use (Bruijnen et al., 2019), substance use (Ramey & Regier, 2019) and cannabis use (Meier et al., 2022) have been strongly associated with cognitive dysfunction. At the same time, our findings suggest that the course of neurocognition in schizophrenia was explicitly linked to the illness and not to comorbid substance use or other risk factors; the association between comorbid substance use and cognitive decline in patients with schizophrenia warrants further research.

#### Long-term neurocognition and correlations of change

We found that higher levels of neurocognition at the 20-year follow-up were significantly associated with better functional outcomes. This is in line with consistent evidence of poor neurocognition being associated with poor outcomes in schizophrenia (Fett et al., 2011; Halverson et al., 2019). However, neurocognition only explained small proportions of the variance (5–21%). Review findings indicate that social cognition may be more strongly associated with poor outcomes, possibly mediating some of the effects of neurocognition on poor outcomes. Furthermore, evidence suggests that negative symptoms mediate the effect of neurocognition on functional outcomes (Glenthøj, Kristensen, Wenneberg, Hjorthøj, & Nordentoft, 2020; Ventura, Hellemann, Thames, Koellner, & Nuechterlein, 2009). Our study found neurocognition to be more strongly associated with negative symptoms than positive psychotic symptoms, and specifically, an increase in experiential negative symptoms significantly correlated with cognitive decline. These findings are in keeping with emerging evidence on the key role of experiential negative symptoms in the cognitive and functional outcome of psychotic disorders (Glenthøj et al., 2020; Glenthøj et al., 2020; Llerena, Reddy, & Kern, 2018). Additionally, it supports including a two-factor model of negative symptoms (expressive and experiential) in clinical studies, as these two aspects may have a differential impact on clinical variables. Speculating, it could be that experiential negative symptoms mediated the effect of cognition on functional outcomes, which would indicate the importance of addressing this negative symptom domain when aiming at improving the cognitive and functional outcome of schizophrenia. Future studies could help clarify this issue.

### Strengths and limitations

The strength of this study was its prospective design and long-term follow-up, along with the large sample size and the representativeness of the OPUS cohort (Petersen et al., 2005b). The main limitation of our study was the significant drop-out rate from the 10 to the 20-year follow-up. Our drop-out analysis indicated that those lost to follow-up may have suffered more severe psychopathology at baseline, meaning there is a risk of an attrition bias in our sample, and in a previous paper, we found that this was true regardless of treatment allocation in the randomized OPUS trial (Hansen et al., 2023b). In addition, drop-out analysis of cognitive score between the 10- and 20-year follow-up revealed that non-participants in the 20-year follow-up had worse cognitive function at the 10-year follow-up. Consequently, our results reflect patients with a better outcome. Those with poor or declining neurocognition were less likely to participate in our follow-up assessments, and it seems possible that poor neurocognition may have been a direct factor hindering follow-up participation. Therefore, our findings may not be representative of all patients with schizophrenia, and we cannot rule out that real-world outcomes are worse than what our findings show. The findings can, however, still be used as evidence of different patterns of neurocognitive trajectories in subgroups of patients with schizophrenia.

Another important limitation was the need for a healthy control group and the lack of BACS normative data for the healthy Danish background population. This limitation means our findings of subgroups with declining, stable, or improving neurocognition cannot be used as firm evidence of different neurocognitive trajectories in schizophrenia compared to healthy controls.

Other limitations include the BACS test, which does not constitute a full cognitive assessment; therefore, we may not have been able to detect a broader range of cognitive deficits corresponding to the MATRICS-defined subdomains. Additionally, no cognitive assessments were conducted at baseline, precluding investigating cognitive changes from illness onset and onwards, and this may also have hindered our ability to identify significant predictors of change.

## Conclusion

Mean neurocognition remained stable from the 10- to the 20-year follow-up. Still, we found that 30.5% of patients had a declining course of neurocognition, 49.2% had a stable course, and 20.3% experienced improvements in neurocognition. These findings support the two existing models of neurocognitive development in schizophrenia and support the growing body of evidence suggesting different subtypes of schizophrenia characterized by different illness phenotypes exist. Unfortunately, because of attrition bias, patients with poorer cognitive function seem underrepresented in our study, and real-life long-term cognitive development might be worse than we could describe. In our patient population, poor long-term cognition was associated with other poor outcomes at the 20-year follow-up, highlighting the importance of treatments aimed at improving neurocognition in patients with schizophrenia spectrum disorders (Lejeune et al., 2021; Wykes et al., 2011).

## Supporting information

Starzer et al. supplementary material 1 Starzer et al. supplementary material

Starzer et al. supplementary material 2 Starzer et al. supplementary material

## Supplementary material

The supplementary material for this article can be found at https://doi.org/10.1017/S0033291724000096.

Supplementary material associated with this article is Appendix A reporting the systematic search strategy and Appendix B containing supplementary tables.
