# Supplementary material for: 20-year neurocognitive development following a schizophrenia spectrum disorder and associations with symptom severity and functional outcomes
Source: Psychol Med. 2024 Feb 12;54(9):2004–14. doi: 10.1017/S0033291724000096 (PMC11413361; doi:10.1017/S0033291724000096)
Supplement: Starzer et al. supplementary material 1 — Starzer et al. supplementary material [file S0033291724000096sup001.docx]

Appendix A, supplementary materials:

Systematic search:

**Search string PubMed:** ((schizophrenia[MeSH Terms]) OR (psychotic disorder[MeSH Terms])) AND ((longitudinal studies[MeSH Terms]) OR (long-term)) AND ((cognition[MeSH Terms]) OR (cognition disorders[MeSH Terms])) – **1076 hits**

**Search string Embase**: Schizophrenia.mp OR psychotic disorder.mp AND neurocognition.mp. OR cognition.mp OR cognitive decline.mp AND long-term.mp OR longitudinal stud* limit to human **– 722 hits**

**Search string PsychInfo**: schizophrenia or psychosis or psychoses or psychotic disorder or schizophrenic disorder AND long-term or longitudinal AND cognition or cognitive function or neurocognition – **1220 hits**

We identified a total of 3018 papers, we deleted 710 duplicates and excluded 2018 based on title alone leaving 290. After reading abstracts we identified 55 papers reporting on longitudinal outcomes of cognition, and we identified 14 papers representing 9 studies that reported on variables associated to level of cognitive functioning after 10 – 25 years. Two studies reported clinical characteristics associated to cross-sectional neurocognitive outcome in first episode psychosis patients, six studies reported clinical characteristics associated to longitudinal changes in neurocognition in first episode patients, and one study reported clinical characteristics associated with changes in neurocognition of chronic schizophrenia patients with a mean age of 55 at study inclusion:

**eTable 1: List of baseline characteristics that have been investigated in association with long-term neurocognitive outcomes:**

| **Baseline** | **No association** | **Association** |
| --- | --- | --- |
| Clinical characteristics that have been examined in association with long-term neurocognition in First episode patients (over 10 years) | Age (2), sex, premorbid social functioning, DUP, use of antipsychotic medication^1^, education, premorbid adjustment, SAPS, SANS, employment, still living with parents, socioeconomic status, diagnosis, substance use, growing up with both parents, diagnosis, living alone, being in a relationship | sex^2^, DAT* (duration of psychosis after treatment), premorbid academic functioning^2^, age at illness onset^2^, employment status^2^, early specialized intervention treatment^2^, finishing highshool^2^, level of education^3^****  negative symptoms^4^ |
| Clinical characteristics that have been examined in association with change in long-term neurocognition in first episode patients (over 10 years) | Age, vocabulary, DUP(2), DAT education, premorbid adjustment, SAPS, SANS, employment, still living with parents, socioeconomic status, diagnosis, substance use, hospitalization, PANSS | Age^5^, DUI^6^***, parents history of mental illness^6^, stable remission**^7^, DAT*****^8^, Baseline neurocognition^9^, level of education^10^, baseline IQ^10^, age of illness onset^10^, schizophrenia diagnosis^11,5^, premorbid IQ^12^, Cannabis sessaition^13^****** symptom severity*******^14^, |
| Variables associated to change in chronic schizophrenia patients | Symptom levels, global functioning, social functioning, disease insight, substance use | Age^1^ |

*Defined as the number of weeks per year with a score of four or higher on PANSS items: P1-delusions, P3-halluciantory behavior, P5-grandiosity, P6-suspiciousness or General scale item 9-unusual thought content. They grouped DAT into three groups of equal numbers of patients termed long, medium and short duration.

**Stable remission in this study was defined as no relapse within the first year after admission

*** Significant only prior to Bonferroni correction

**** divided into low (up to 8 years) medium (8-12 years) and high (longer than 12 years), and subdivided age into low and high below or above 10 years)

***** They defined the three groups (short, medium and long) by dividing them into equal sizes

****** They used self-reported use of cannabis to divide participants into four groups based on patterns of use: Non-users patients who had never used, b) stop-users, c) episodic users, and d) persistent users. Patients who had only “no-use” measurements during the first 2 years of follow-up were defined as nonusers (NUs). Patients who had used at baseline and then not use for at least two consecutive measurements, i.e.  at 1 and 2 years of follow-up, were defined as stop-users (SUs). Persistent users (PUs) used at all follow-up points, and episodic users (EUs) had various other substance-use patterns.

******* They used SCAN to index symptom severity into 4 groups: 0=absent, 1=mild, 2=moderate, and 3=severe

Selection of predictors of neurocognitive change for our analysis

**Variables associated with poor long-term neurocognition were:**

1) male sex, 2) younger age at illness onset, 3) unemployment, 4) not finishing high-school 5) lower level of education and 6) premorbid academic functioning and 7) not receiving early specialised interventions. We combined not finishing high-school and lower level of education into one variable dividing patients into two groups with above or below 10 years of education.

**Variables associated with changes in neurocognition were**:

1. Age^5^, 2) Duration of untreated illness^6^, 3) parents history of mental illness^6^, 4) stable remission in the first year^7^, 5) duration of psychosis after treatment initiation^8^, 6) baseline neurocognition^9^, 7) level of education^10^, 8) baseline IQ^10^, 9) age at illness onset^10^, 10) a schizophrenia diagnosis^11,5^, 11) premorbid IQ^12^, 12) cannabis sessaition^13^, and 13) indexed symptom severity^14^.

To match the identified variables, we had to create new variables from our own data. To create a variable expressing symptom remission in the first year, we combined psychotic symptom assessments conducted every three months during the first two years of the OPUS trial. Because they were assessed using SAPS, they covered all three months between every assessment, allowing us to create a variable of continuous remission during the first year following diagnosis for all patients that did not exhibit significant psychotic symptoms following their first admission in the study. The study on cessation of cannabis use used cessation between baseline and the end of their study, which is when they conducted their cognitive tests. For this reason, we did not include baseline cannabis use in our analysis, but the pattern of use during the 10-and 20-year follow-up, which is when we conducted our cognitive testing. Unfortunately, our sample was too small to examine strict cessation of cannabis use, as this only occurred in 4 patients. eTable 2 shows the distribution of patterns of substance use between the 10- and 20-year follow-up. Instead, we have chosen overall substance and alcohol abuse diagnosed using SCAN as a variable in this study.

eTable 2: Patterns of substance use between the 10- and 20-year follow-up, Appendix 1:

|  | **No use** | **Continued use** | **Started use** | **Stopped use** |
| --- | --- | --- | --- | --- |
| Overall alcohol or substance use | 115 (75.2%) | 23 (15%) | 8 (5.2%) | 7 (4.6%) |
| Alcohol use | 132 (86.3%) | 9 (5.9%) | 5 (3.3%) | 7 (4.6%) |
| Cannabis use | 138 (90.2%) | 8 (5.2%) | 3 (2.0%) | 4 (2.6%) |
| Other groups (opioids, cocaine, mixed) | 146 (95.4%) | 1 (0.7%) | 5 (3.3%) | 1 (0.7%) |

We did not have information on parents history of mental illness^6^, duration of psychosis after treatment initiation^8^, baseline neurocognition^9^ and baseline IQ (except it had to be over 70 for inclusion in the study). We also did not have information un duration of untreated illness an chose to use duration of untreated psychosis instead. Because of collinearity between indexed symptom severity and the symptom remission categorical variable we only included stable remission in our analysis, and because of collinearity of premorbid academic functioning and premorbid IQ, we chose only to include premorbid academic functioning in our analysis, as we had more data on this variable.

**References:**

1. Meier MH, Caspi A, Reichenberg A, et al. Neuropsychological decline in schizophrenia from the premorbid to the postonset period: Evidence from a population-representative longitudinal study. *Am J Psychiatry*. 2014;171(1):91-101. doi:10.1176/APPI.AJP.2013.12111438/ASSET/IMAGES/LARGE/91F2.JPEG

2. Bergh S, Hjorthøj C, Sørensen HJ, et al. Predictors and longitudinal course of cognitive functioning in schizophrenia spectrum disorders, 10 years after baseline: The OPUS study. *Schizophr Res*. 2016;175(1-3):57-63. doi:10.1016/J.SCHRES.2016.03.025

3. Ayesa-Arriola R, Miguel-Corredera M, De La Foz VOG, et al. Education and long-term outcomes in first episode psychosis: 10-year follow-up study of the PAFIP cohort. *Psychol Med*. 2023;53(1):66-77. doi:10.1017/S0033291721001112

4. Luther L, Suor JH, Rosen C, Jobe TH, Faull RN, Harrow M. Clarifying the direction of impact of negative symptoms and neurocognition on prospective work functioning in psychosis: A 20-year longitudinal study. *Schizophr Res*. 2020;220:232-239. doi:10.1016/J.SCHRES.2020.03.012

5. Fett AKJ, Velthorst E, Reichenberg A, et al. Long-term Changes in Cognitive Functioning in Individuals with Psychotic Disorders: Findings from the Suffolk County Mental Health Project. *JAMA Psychiatry*. 2020;77(4):387-396. doi:10.1001/jamapsychiatry.2019.3993

6. Rodríguez-Sánchez JM, Setién-Suero E, Suárez-Pinilla P, et al. Ten-year course of cognition in first-episode non-affective psychosis patients: PAFIP cohort. *Psychol Med*. 2022;52(4):770-779. doi:10.1017/S0033291720002408

7. Rund BR, Barder HE, Evensen J, et al. Neurocognition and Duration of Psychosis: A 10-year Follow-up of First-Episode Patients. *Schizophr Bull*. 2016;42(1):87-95. doi:10.1093/SCHBUL/SBV083

8. Barder HE, Sundet K, Rund BR, et al. 10 year course of IQ in first-episode psychosis: Relationship between duration of psychosis and long-term intellectual trajectories. *Psychiatry Res*. 2015;225(3):515-521. doi:10.1016/J.PSYCHRES.2014.11.054

9. Hoff AL, Svetina C, Shields G, Stewart J, DeLisi LE. Ten year longitudinal study of neuropsychological functioning subsequent to a first episode of schizophrenia. *Schizophr Res*. 2005;78(1):27-34. doi:10.1016/J.SCHRES.2005.05.010

10. Murillo-García N, Ortíz-García de la Foz V, Miguel-Corredera M, et al. Intelligence quotient changes over 10 years: Diversity of cognitive profiles in first episode of psychosis and healthy controls. *Schizophr Res*. 2023;254:163-172. doi:10.1016/J.SCHRES.2023.02.025

11. Jonas K, Lian W, Callahan J, et al. The Course of General Cognitive Ability in Individuals With Psychotic Disorders. *JAMA Psychiatry*. 2022;79(7):659-666. doi:10.1001/JAMAPSYCHIATRY.2022.1142

12. van Winkel R, Myin-Germeys I, Delespaul P, Peuskens J, De Hert M, van Os J. Premorbid IQ as a predictor for the course of IQ in first onset patients with schizophrenia: A 10-year follow-up study. *Schizophr Res*. 2006;88(1-3):47-54. doi:10.1016/J.SCHRES.2006.06.033

13. Weibell MA, Johannessen JO, Auestad B, et al. Early Substance Use Cessation Improves Cognition-10 Years Outcome in First-Episode Psychosis Patients. *Front psychiatry*. 2019;10(JULY). doi:10.3389/FPSYT.2019.00495

14. Zanelli J, Mollon J, Sandin S, et al. Cognitive Change in Schizophrenia and Other Psychoses in the Decade Following the First Episode. *Am J Psychiatry*. 2019;176(10):811-819. doi:10.1176/APPI.AJP.2019.18091088
