# Supplementary material for: 20-year neurocognitive development following a schizophrenia spectrum disorder and associations with symptom severity and functional outcomes
Source: Psychol Med. 2024 Feb 12;54(9):2004–14. doi: 10.1017/S0033291724000096 (PMC11413361; doi:10.1017/S0033291724000096)
Supplement: Starzer et al. supplementary material 2 — Starzer et al. supplementary material [file S0033291724000096sup002.docx]

# Appendix B, supplementary material

**eTable 1: Drop-out analysis of significant differences between participants and non-participants in the 20-year reassessment:**

|  | Total  578 | Participants  156 | Nonparticipants:  422 | Significant difference (p-value) |
| --- | --- | --- | --- | --- |
| Characteristics | Number with (%) or mean with (SD) | | | |
| **Age (mean)** | **26.8 (SD 6.5)** | **25.7 (SD 5.7)** | **26.9 (SD 6.5)** | **0.021** |
| **Male (number)** | **343 (59.3)** | **76 (48.7%)** | **267 (63.3%)** | **0.002** |
| Employment | 178 (30.8%) | 57 (36.5%) | 121 (28.7%) | 0.084 |
| **Over 10 years of education** | **191 (33.5%)** | **68 (43.6%)** | **123 (29.7%)** | **0.002** |
| Independent living | 534 (92.4%) | 147 (94.2%) | 387 (91.7%) | 0.379 |
| In a relationship | 139 (24.4%) | 39 (25%.0%) | 100 (24.2%) | 0.828 |
| Premorbid social functioning * | 0.76 (SD 0.59) | 0.76 (SD 0.58) | 0.76 (SD 0.6) | 0.987 |
| Premorbid academic functioning * | 1.00 (SD 0.63) | 0.94 (SD 0.6) | 1.02 (SD 0.64) | 0.182 |
| OPUS Treatment | 275 (47.6%) | 77 (49.4%) | 198 (46.9%) | 0.695 |
| Schizophrenia diagnosis (number) | 388 (67.1%) | 97 (62.2%) | 291 (69.0%) | 0.135 |
| **Schizotypal diagnosis** | **82 (14.2%)** | **30 (19.2%)** | **52 (12.3%)** | **0.043** |
| Substance use disorder | 162 (28%) | 40 (25.6%) | 122 (28.9%) | 0.467 |
| Psychopathology— mean (S.D.) | | | | |
| **Negative dimension** | **2.19 (SD 1.16)** | **1.9 (SD 1.1)** | **2.3 (SD 1.2)** | **0.001** |
| Psychotic dimension | 2.72 (SD 1.46) | 2.6 (SD 1.5) | 2.8 (SD 1.4) | 0.202 |
| Duration of untreated psychosis (DUP) – in weeks | Mean 112.4 (SD 187)  Median 50.43  Range (13.0 – 156.4) | 128.5 (SD 215.1) | 106.6 (SD 175.9) | Mann Whitney U  p = 0.475 |
| Age at onset of illness | 24.4 (SD 6.3) | 23.6 (SD 6.0) | 24.7 (SD6.4) | 0.090 |
| **Global assessment of functioning (GAF) – mean** | **40.1 (SD 13.2)** | **44.2 (SD 13.2)** | **39.7 (SD 13.1)** | **<0.001** |

**eTable 2: Sociodemographic outcomes at the 10- and 20-year follow-up:**

|  | | **10 years (n 367)**  **N and %** | | **20 years (n 174)**  **Mean and SD** | |
| --- | --- | --- | --- | --- | --- |
| **Sociodemographic variables, n (%)** | | | | | |
| Age, mean (SD) | | 36.96 (6.45) | | 45.90 (5.45) | |
| Sex, female | | 161 (43.9%) | | 87 (50.0%) | |
| Independent living | | 246 (94.5%) | | 163 (93.7%) | |
| Married | | 61 (16.6%) | | 30 (17.2%) | |
| Homeless at any point during last 2 years | | 10 (2.8%) | | 3 (1.7%) | |
| Employed | | 90 (24.7%) | | 52 (29.9%) | |
| Use of antipsychotic medication | | 212 (58.2%) | | 89 (51.1%) | |
| Use of benzodiazepines | | 28 (7.6%) | | 13 (7.5%) | |
| Received OPUS treatment | | 181 (49.3%) | | 84 (48.3%) | |
| **Diagnosis** | | | | | |
| Schizophrenia | | 289 (78.7%) | | 103 (59.2%) | |
| Schizotypal disorder | | 37 (10.1%) | | 26 (14.9%) | |
| Other psychosis (F22-F29) | | 41 (11.2%) | | 6 (3.5%) | |
| Substance and/or alcohol misuse | | 98 (26.7%) | | 34 (19.5%) | |
| Cannabis abuse | | 38 (10.4%) | | 13 (7.5%) | |
| Alcohol abuse | | 51 (13.9%) | | 15 (8.6%) | |
| Other substances (opioids, cocaine, mixed) | | 7 (1.9%) | | 6 (3.4%) | |
| **Changes in psychopathology from 10- to 20-year follow-up (n 153 participated in both) Compared using paired sample t-test** | | | | | |
|  | 10 year follow-up  Mean and SD | | **20 year follow-up Mean and SD** | | **P value** |
| **Social and global functioning** | | | | | |
| Global function (GAF) (n 153) | 59.1 (15.9) | | 57.4 (15.4) | | <0.001 |
| Social functioning (PSP) (n 153) | 59.3 (16.0) | | 57.3 (15.7) | | 0.057 |
| **Neurocognitive function, BACS raw scores** | | | | | |
| Verbal memory task (n 141) | 45.3 (13.5) | | 44.4 (10.8) | | 0.375 |
| Number sequencing task (n 140) | 18.8 (5.2) | | 18.3 (4.6) | | 0.118 |
| Token motor task (n 138) | 68.1 (12.6) | | 68.9 (17.6) | | 0.537 |
| Fluency task (n 140) | 56.9 (16.8) | | 56.3 (16.4) | | 0.575 |
| Symbol coding task (140) | 47.7 (13.8) | | 50.8 (14.7) | | <0.001 |
| Tower of London (n 137) | 18 (3.1) | | 17.9 (3.4) | | 0.711 |
| **Psychopathology** | | | | | |
| Psychotic dimension (n 153) | 0.95 (1.4) | | 1.12 (1.4) | | 0.075 |
| Disorganised dimension (n 150) | 0.41 (0.1) | | 0.67 (0.1) | | <0.001 |
| Negative dimension (n 153) | 1.25 (1.0) | | 1.56 (1.1) | | <0.001 |
| - Experiential symptom subdomain | 0.93 (0.9) | | 1.41 (1.0) | | <0.001 |
| - Expressive symptom subdomain | 0.57 (0.7) | | 0.66 (0.9) | | 0.226 |

**eTable 3: Drop-out analysis of differences in 10-year cognitive function between participants and non-participants in the 20-year follow-up**

|  | **Total** | **Participants** | **Non-participants** | **P-value** |
| --- | --- | --- | --- | --- |
| **Global cognition** | 0 (SD 1) | 0.24 (SD 1.33) | -0.17 (0.63) | <0.001 |

**eTable 4: Analysis of 20-year outcomes associated with 20-year neurocognition subdomain z-scores:**

| **Bivariate logistic regression of categorical outcomes** | | | | | | | | | | | | | | | | | |
| --- | --- | --- | --- | --- | --- | --- | --- | --- | --- | --- | --- | --- | --- | --- | --- | --- | --- |
|  | **In a relationship** | | | | **Employment** | | | **Independent living** | | | **Schizophrenia diagnosis** | | | | **Use of antipsychotic medication** | | |
| **Cognitive Z scores increasing** | **Odds ratio and (CI)** | | **P-value** | | **Odds ratio and (CI)** | | **P-value** | **Odds ratio and (CI)** | | **P-value** | **Odds ratio and (CI)** | | **P-value** | | **Odds ratio and (CI)** | | **P-value** |
| **Verbal learning and memory** | 1.46 (1.05:2.04) | | 0.024 | | 1.94 (1.31:2.88) | | <0.001 | 2.10 (0.63:6.98) | | 0.227 | 0.57 (0.40:0.80) | | 0.001 | | 0.37 (0.25:0.56) | | <0.001 |
| **Speed of processing** | 1.41 (1.01:1.97) | | 0.041 | | 1.93 (1.29:2.89) | | <0.001 | 1.85 (0.7:4.92) | | 0.215 | 0.48 (0.32:0.66) | | <0.001 | | 0.59 (0.42:0.84) | | 0.003 |
| **Executive function** | 1.53 (1.05:2.23) | | 0.026 | | 1.47 (0.98:2.20) | | 0.066 | 1.67 (0.62:4.53) | | 0.314 | 0.90 (0.64:1.26) | | 0.531 | | 0.60 (0.41:0.87) | | 0.007 |
| **Linear regression analysis of numerical outcomes** | | | | | | | | | | | | | | | | | |
|  | | **Social functioning** | | | | | **Psychotic symptoms** | | | | | **Negative symptoms** | | | | | |
| **Cognitive Z scores increasing** | | **B (95% CI)** | | √**R** | | **p-value** | **B (95% CI)** | | √**R** | **p-value** | | **B (95% CI)** | | √**R** | | **p-value** | |
| **Verbal learning and memory**  **(n 160)** | | 6.77 (4.65:8.88) | | 0.20 | | <0.001 | -0.21  (-0.42:-0.01) | | 0.02 | 0.055 | | -0.52  (-0.68:-0.36) | | 0.22 | | <0.001 | |
| **Speed of processing (n 159)** | | 6.26 (4.07:8.46) | | 0.17 | | <0.001 | -0.27  (-0.49:-0.05) | | 0.04 | 0.015 | | -0.43  (-0.6:-0.27) | | 0.15 | | <0.001 | |
| **Executive function (n 160)** | | 4.06 (1.61:6.52) | | 0.06 | | 0.001 | -0.08 (-0.31:0.15) | | 0.00 | 0.472 | | -0.19  (-0.38:-0.0) | | 0.03 | | 0.046 | |

**eTable 5: Pearson correlation analysis of correlations between change in cognition subdomains and change in symptom severity and social functioning**

|  | **Verbal learning and memory change scores (n 139)** | | **Speed of processing change scores (n 135)** | | **Executive functioning change scores (n 137)** | |
| --- | --- | --- | --- | --- | --- | --- |
|  | **Pearsons correlation**  **and (CI)** | **p** | **Pearsons correlation**  **and (CI)** | **p** | **Pearsons correlation**  **and (CI)** | **p** |
| **Positive symptom change scores** | -0.07 (-0.23:0.10) | 0.437 | 0.09  (-0.08:0.26) | 0.301 | 0.06 (-0.11:0.23) | 0.491 |
| **Negative symptom change scores** | **-0.18 (-0.34:-0.01)** | **0.038** | **-0.19**  **(-0.35:-0.02)** | **0.026** | 0.14 (-0.03:0.3) | 0.117 |
| ***- Experiential symptom change scores*** | *-0.18 (-0.36:0.02)* | *0.077* | *-0.12*  *(-0.32:0.08)* | *0.231* | *0.02 (-0.19:0.21)* | *0.888* |
| ***- Expressive symptom change scores*** | *-0.15 (-0.31:0.02)* | *0.081* | *-0.03*  *(-0.2:0.15)* | *0.761* | *0.01 (-0.16:0.18)* | *0.904* |
| **Social functioning change scores** | 0.15 (-0.02:0.31) | 0.083 | 0.16  (-0.01:0.32) | 0.060 | 0.13 (-0.4:0.29) | 0.125 |

**eTable 6:** **Univariate linear regression analysis (ANCOVA) of baseline characteristics associated with changes in cognitive subdomains:**

| **Univariate ANCOVA analysis (significance set at p=0.01)** | | | | | | |
| --- | --- | --- | --- | --- | --- | --- |
|  | **Changes from the 10- to the 20-year follow-up** | | | | | |
| **Baseline characteristics** | **Change in verbal learning and memory (n 139)** | | **Change in speed of processing (n 135)** | | **Change in executive function (n 137)** | |
|  | **B (95% CI)** | **p-value** | **B (95% CI)** | **p-value** | **B (95% CI)** | **p-value** |
| **Male sex** | 0.001  (-0.217:0.214) | 0.989 | -0.075  (-0.353:0.203) | 0.594 | -0.129  (-0.149:0.407) | 0.361 |
| **Age (increasing)** | -0.014  (-0.032:0.005) | 0.155 | 0.011  (-0.013:0.035) | 0.364 | -0.011  (-0.035:0.014) | 0.338 |
| **Unemployment** | 0.010  (0.235:-0.214) | 0.929 | -0.228  (0.059:-0.514) | 0.118 | -0.065  (0.229:-0.358) | 0.664 |
| **Lower level of education** | -0.045  (0.172:-0.261) | 0.684 | -0.238  (-0.516:0.041) | 0.094 | 0.115  (-0.165:0.395) | 0.420 |
| **Lower premorbid academic functioning** | 0.083  (-0.100:0.266) | 0.371 | 0.125  (0.355:-0.105) | 0.284 | -0.051  (0.184:-0.286) | 0.666 |
| **OPUS** | -0.36  (0.081:-0.154) | 0.542 | 0.049  (0.216:-0.119) | 0.567 | -0.026  (0.134:-0.185) | 0.749 |
| **Stable remission first year** | -0.001  (0.241:-0.243) | 0.993 | -0.051  (0.258:-0.359) | 0.746 | 0.286  (0.596:-0.024) | 0.070 |
| **Higher age at illness onset** | -0.005  (0.013:-0.023) | 0.552 | 0.016  (0.039:-0.007) | 0.170 | -0.017  (0.006:-0.040) | 0.150 |
| **Longer duration of untreated psychosis (weeks)** | 0.000  (-0.001:0.000) | 0.154 | 0.000  (0.000:-0.001) | 0.301 | 0.001 (0.000:0.002) | 0.186 |
| **Schizophrenia diagnosis** | -0.013 (-0.076:0.050) | 0.686 | -0.024 (-0.105:0.057)  (-0.280:0.228) | 0.559 | -0.038 (-0.119:0.043)  (-0.2030.367) | 0.356 |
| **Cessation of alcohol or substance use including cannabis** | -0.312  (-0.840:0.215) | 0.244 | 0.194  (-0.431:0.820) | 0.540 | -0.066  (-0.698:567) | 0.837 |
